# The Proteome Signatures of Fibroblasts from Patients with Severe, Intermediate and Mild Spinal Muscular Atrophy Show Limited Overlap

**DOI:** 10.3390/cells11172624

**Published:** 2022-08-23

**Authors:** Sharon J. Brown, Rachel A. Kline, Silvia A. Synowsky, Sally L. Shirran, Ian Holt, Kelly A. Sillence, Peter Claus, Brunhilde Wirth, Thomas M. Wishart, Heidi R. Fuller

**Affiliations:** 1 School of Pharmacy and Bioengineering (PhaB), Keele University, Keele ST5 5BG, UK; 2 Wolfson Centre for Inherited Neuromuscular Disease, RJAH Orthopaedic Hospital, Oswestry SY10 7AG, UK; 3 The Roslin Institute and Royal (Dick) School of Veterinary Studies, University of Edinburgh, Midlothian EH25 9RG, UK; 4 Euan MacDonald Centre, University of Edinburgh, Edinburgh EH16 4SB, UK; 5 BSRC Mass Spectrometry and Proteomics Facility, University of St Andrews, St Andrews KY16 9ST, UK; 6 Bio-Rad Laboratories, Watford WD17 1ET, UK; 7 SMATHERIA gGmbH—Non-Profit Biomedical Research Institute, 30625 Hannover, Germany; 8 Institute of Human Genetics, University Hospital of Cologne, University of Cologne, 50931 Cologne, Germany; 9 Center for Rare Diseases, University Hospital of Cologne, University of Cologne, 50931 Cologne, Germany; 10 Center for Molecular Medicine Cologne, University of Cologne, 50931 Cologne, Germany; 11 Institute for Genetics, University of Cologne, 50931 Cologne, Germany

**Keywords:** Spinal muscular atrophy, SMA, fibroblasts, severity, proteomics, biomarkers, SMN, RAB3B

## Abstract

Most research to characterise the molecular consequences of spinal muscular atrophy (SMA) has focused on SMA I. Here, proteomic profiling of skin fibroblasts from severe (SMA I), intermediate (SMA II), and mild (SMA III) patients, alongside age-matched controls, was conducted using SWATH mass spectrometry analysis. Differentially expressed proteomic profiles showed limited overlap across each SMA type, and variability was greatest within SMA II fibroblasts, which was not explained by *SMN2* copy number. Despite limited proteomic overlap, enriched canonical pathways common to two of three SMA severities with at least one differentially expressed protein from the third included mTOR signalling, regulation of eIF2 and eIF4 signalling, and protein ubiquitination. Network expression clustering analysis identified protein profiles that may discriminate or correlate with SMA severity. From these clusters, the differential expression of PYGB (SMA I), RAB3B (SMA II), and IMP1 and STAT1 (SMA III) was verified by Western blot. All SMA fibroblasts were transfected with an SMN-enhanced construct, but only RAB3B expression in SMA II fibroblasts demonstrated an SMN-dependent response. The diverse proteomic profiles and pathways identified here pave the way for studies to determine their utility as biomarkers for patient stratification or monitoring treatment efficacy and for the identification of severity-specific treatments.

## 1. Introduction

The inherited neuromuscular disease spinal muscular atrophy (SMA) is primarily characterised by loss of lower motor neurons and subsequent muscular atrophy [1,2]. Preclinical studies in patients and animal models have highlighted systemic aspects of the disease, which have recently been reviewed [3] and include, for example, impairment of skeletal muscle development [4], cardiac defects and dysfunction [5,6], detrimental vascular changes [7], defects in fatty acid metabolism [8] and altered renal structure and function [9,10]. In more than 95% of cases, SMA is caused by loss of function of the *survival of motor neuron 1* (*SMN1*) gene, resulting in insufficient levels of SMN protein [11]. Most humans possess at least one copy of an additional *SMN2* gene, which is almost identical to *SMN1*, but the protein translated from it is far less stable and unable to compensate entirely for the loss of *SMN1* [12,13]. Although *SMN2* copy number has some bearing on the phenotypic outcome, with an increased copy number generally predicting a less severe SMA phenotype, this is not the case for all SMA patients, and there remain outstanding questions regarding modifiers of SMA severity [14,15].

Traditionally, SMA is classified into five clinical sub-types depending on the developmental milestones reached: Type 0 (severe neonatal), Type I (severe), Type II (intermediate), Type III (mild) and Type IV (adult-onset) [1,2]. Current treatments in the clinic are focused on restoring SMN protein levels in patients either by delivering cDNA of *SMN1* using a viral vector (AAV9) to the cells (Onasemnogene abeparvovec) or by manipulating *SMN2* mRNA splicing so that exon 7 is included (Nusinersen and Risdiplam), thus upregulating functional protein production [16]. These treatments have varied success and are beginning to alter the established natural progression of SMA, resulting in the generation of new phenotypes that span the typical sub-types of SMA [17].

Preclinical and clinical studies have shown that SMN has an important role during development, suggesting that increasing SMN levels after birth may not be sufficient to successfully treat every aspect of the disease. It is known, for example, that reduced SMN levels in neonatal mice result in an SMA-like phenotype, but depletion of SMN in adult mice has relatively little effect [18,19]. The timing of insensitivity to low SMN coincides with full maturation of the motor unit [19], and in line with this, restoration of SMN after this time fails to produce a therapeutic benefit in SMA mice [18,20,21]. From a study of an “intermediate” mouse model of SMA, a critical threshold has been established, in terms of the amount of SMN protein that is expressed, that determines the onset of SMA [22]. Decreasing SMN levels in this mouse model by just 15% of the normal level resulted in reduced body weight, motor neuron loss and motor defects. Studies of the Taiwanese mouse model of severe SMA have also found evidence to suggest that reduced SMN levels impact other developmental processes, including lung, intestine, heart and longitudinal bone growth [23,24,25]. Together, these studies suggest that if the level of SMN protein does not meet the threshold required for healthy development in SMA patients, some systems, including, for example, the entire motor unit, could well be dysfunctional even before birth. Conversely, it is conceivable that patients with less severe forms of SMA may express sufficient levels of SMN for appropriate development and that some degenerative pathways that subsequently occur could be quite different to those that are triggered by developmental defects in Type I SMA.

A key issue in our understanding of SMA at present, however, is that almost all preclinical work has focused on identifying and characterising molecular pathways in cells from severe SMA patients (i.e., Type I) and relatively severe mouse models of SMA. It is unclear, for example, whether molecular pathways implicated in SMA Type I are also relevant to SMA Types II and III and, consequently, whether therapeutic strategies and molecular biomarkers for patient stratification and/or therapeutic monitoring would translate effectively across the different types and severities of SMA. The aim of this work, therefore, was to quantitatively compare the proteomic profiles of skin fibroblasts from patients with Type I, Type II and Type III SMA to identify whether a core molecular response to reduced SMN is conserved across the different types of SMA, and then to highlight detectable molecular differences that exist between SMA type and severity.

## 2. Results

### 2.1. Fibroblasts from SMA II Patients Have Greater Variability in Their Proteomic Profiles Compared to Fibroblasts from Patients with SMA I and III

**Figure 1:**
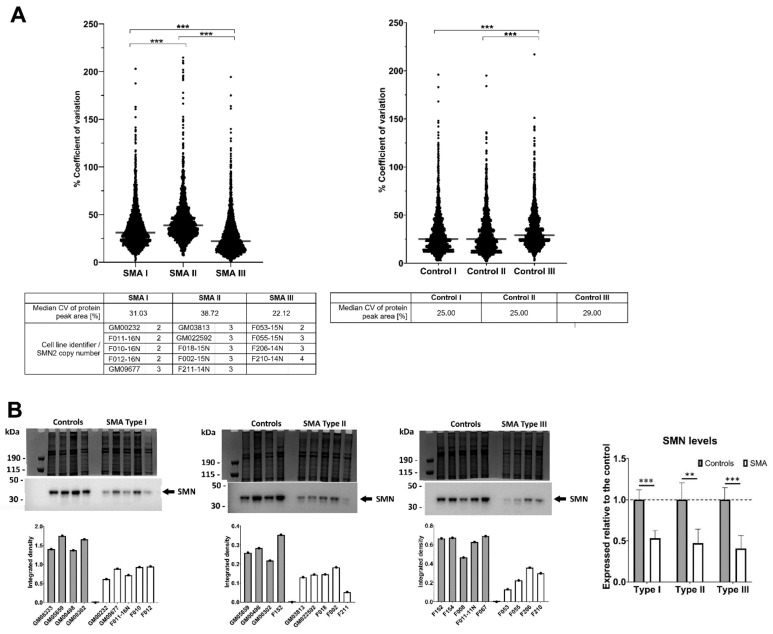
Variability in protein expression and levels of SMN protein in SMA Types I, II and III fibroblasts and respective age-matched controls. (**A**) Summary of the coefficients of variation for the peak areas of all proteins identified following SWATH mass spectrometry for each SMA group and corresponding age-matched controls. Bars indicate the median values within each SMA and control group, which are detailed in the tables below. For each SMA group, the cell line identifier and associated *SMN2* copy number are also listed. *** *p* < 0.0001. (**B**) Expression levels of total SMN protein as determined by Western blot and Image J analysis. The graphs below the Western blots illustrate the integrated density of antibody-reactive bands/densitometry measurements of a Coomassie-stained gel (i.e., total protein loading control) for individual samples. The graph entitled “SMN levels” provides the average SMN level in each SMA type expressed relative to their respective controls, with a dashed line indicating normalised SMN levels across all control fibroblasts. Error bars show the standard deviation from the mean. *** *p* < 0.001; ** *p* < 0.01.

To determine whether the proteomic profiles of fibroblasts differ between patients with different severities of SMA, a quantitative proteomic comparison with age-matched control fibroblasts (Supplementary Materials (Table S1)) was undertaken using Sequential Window Acquisition of all Theoretical Mass Spectra (SWATH-MS). This approach identified 2834 proteins in total (Supplementary Table S3). The extent of variability between proteomic profiles of patients within each SMA group was established by comparing the coefficient of variation of all protein peak areas. Compared to fibroblasts from SMA I and SMA III patients, SMA II fibroblasts had greater variability in their proteomic profiles between patients (*p* < 0.0001), which could not be explained by *SMN2* copy number, as all SMA II patients carried three *SMN2* copies (Figure 1A). Whilst the SMA II fibroblasts came from donors with a wider range of ages than the SMA I fibroblasts (1 year–25 years and 4 months–2 years, respectively; Supplementary Materials (Table S1)), this does not entirely explain the difference in variation either, since the median coefficients of variation for the age-matched controls for SMA I (5 months–3 years) and II (10 months to 27 years) were both 25% (Figure 1A). Despite having the most variability in *SMN2* copy number (ranging from two–four copies) and the widest range of ages (17 years–66 years), SMA III fibroblasts showed a statistically significantly lower level of variation in the proteomic profiles between patients compared to SMA I and II fibroblasts (*p* < 0.0001) (Figure 1A). The expression levels of total SMN protein were consistently reduced in patient fibroblasts of all SMA severities relative to their respective age-matched controls (i.e., SMA I/control = 0.53, *p* < 0.001; SMA II/control = 0.47, *p* = 0.006; and SMA III/control = 0.41, *p* = 0.001), but these levels did not, however, differentiate between SMA types (Figure 1B). This is perhaps not surprising, since a similar observation was noted in a study of SMA patient peripheral blood mononuclear cells [26].

### 2.2. Differentially Expressed Proteomic Profiles of SMA I, II and III Patient Fibroblasts Show Little Overlap

**Figure 2:**
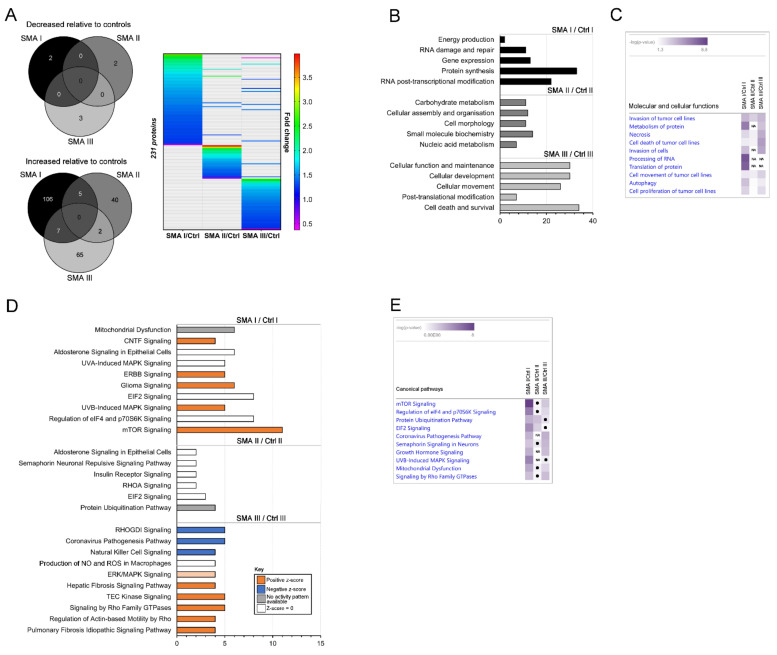
Summary and bioinformatics analysis of proteomic profiles of SMA Types I, II and III fibroblasts relative to their age-matched controls. (**A**) Venn diagrams illustrating the lack of overlap between significantly downregulated and upregulated proteins in each SMA type and heat map detailing the extent of the fold changes relative to age-matched controls. (**B**) Bar charts showing the top five significantly enriched molecular and cellular functions relating to the differentially expressed proteins in each SMA type following Ingenuity Pathway Analysis (IPA^®^). The *x*-axis relates to the number of dysregulated proteins associated with each function. (**C**) A three-way comparison generated in IPA^®^ in which the enriched cellular and molecular functions are ranked by −log(*p*-value) for each SMA type. “NA” denotes terms for which no differentially expressed proteins were associated within the specified dataset. (**D**) Bar charts showing the top ten significantly enriched canonical pathways for the SMA Type I and III fibroblasts (also ranked by number of molecules) and the enriched canonical pathways for SMA Type II fibroblasts after excluding terms matched with only one molecule. The *x*-axis relates to the number of dysregulated proteins associated with each function. (**E**) A three-way comparison generated in IPA^®^ in which the enriched canonical pathways are ranked by −log(*p*-value) for each SMA type. Black dots denote terms that were not enriched with statistical significance (i.e., *p* ≥ 0.05) but that had at least one differentially expressed protein associated with it, and “NA” denotes terms for which no differentially expressed proteins were associated within the specified dataset.

For reliable quantification, proteins identified in the SWATH-MS analysis from just a single peptide were removed, after which differentially expressed proteins were identified by the exclusion of proteins with a fold change that was >0.80 but <1.25 (i.e., less than 25% change in expression) and, finally, the exclusion of proteins with a *p*-value of >0.05 assigned to their fold changes (Supplementary Table S4). When compared to age-matched controls for each SMA type (as described in Supplementary Materials (Table S1)), differentially expressed protein profiles comprised 120 proteins in the SMA I fibroblasts, 49 proteins in the SMA II fibroblasts and 77 proteins in the SMA III fibroblasts (Figure 2A and Supplementary Table S4). None of the differentially expressed proteins consistently met the criteria for differential expression across all SMA types compared to their respective age-matched controls, but some proteins did meet the criteria for increased expression in two out of the three comparisons (i.e., five proteins in SMA I and II, seven proteins in SMA I and III and two proteins in SMA II and III (Figure 2A)).

Ingenuity Pathway Analysis (IPA^®^) highlighted an enrichment of molecular and cellular functions relating to the differentially expressed proteins in each SMA type compared to their respective age-matched controls (Figure 2B). In SMA I, the top five enriched terms were associated with energy production (*p* = 2.44 × 10^−5^), RNA damage and repair (*p* = 2.00 × 10^−3^–1.06 × 10^−5^), gene expression (*p* = 4.96 × 10^−3^–1.06 × 10^−5^), protein synthesis (*p* = 8.33 × 10^−4^–2.57 × 10^−8^) and RNA post-transcriptional modification (*p* = 9.89 × 10^−3^–1.13 × 10^−8^). For SMA II, the top enriched annotations were carbohydrate metabolism (*p* = 4.47 × 10^−2^–2.71 × 10^−4^), cellular assembly and organisation (*p* = 4.54 × 10^−2^–1.63 × 10^−4^), cell morphology (*p* = 4.36 × 10^−2^–1.63 × 10^−4^), small molecular biochemistry (*p* = 4.91 × 10^−2^–5.46 × 10^−5^) and nucleic acid metabolism (*p* = 1.92 × 10^−2^–5.46 × 10^−5^), but fewer differentially expressed proteins were associated with the enriched terms compared to SMA I and III, possibly reflecting the lower number of differentially expressed proteins consistently identified across this group. The top enriched terms in the SMA III dataset included cellular function and maintenance (*p* = 9.43 × 10^−3^–2.94 × 10^−5^), cellular development (*p* = 1.01 × 10^−2^–2.94 × 10^−5^), cellular movement (*p* = 1.08 × 10^−2^–1.60 × 10^−5^), post-translational modification (*p* = 6.29 × 10^−3^–9.80 × 10^−6^) and cell death and survival (*p* = 9.69 × 10^−3^–6.95 × 10^−6^). A three-way comparison in IPA^®^ in which the enriched “cellular and molecular functions” annotations across each SMA type were ranked by absolute *p*-value indicated that there is very little overlap of enrichment per comparison (SMA type vs. control) (Figure 2C). While terms associated with tumour cell lines showed some degree of overlap between SMA types, these are likely unrelated to SMA, instead reflecting the high dominance of cancer-related studies constituting the protein–protein and protein–DNA interactions comprising the IPA^®^ Knowledge Base, especially since tumour-related terms were not among the most enriched terms when the SMA comparisons were analysed in isolation (Figure 2B). After tumour-related terms were excluded, the only terms with some overlap were necrosis (SMA I (*p* = 1.22 × 10^−3^), SMA II (*p* = 9.93 × 10^−3^) and SMA III (*p* = 2.8 × 10^−5^)), autophagy (SMA I (*p* = 1.75 × 10^−4^), SMA II (*p* = 3.08 × 10^−3^) and SMA III (*p* = 7.96 × 10^−3^)), and metabolism of protein (SMA 1 (*p* = 3.72 × 10^−7^) and SMA III (*p* = 2.05 × 10^−4^) only) (Figure 2C).

Having established profiles of differentially expressed proteins in each SMA type compared to their respective controls and having shown that there is limited overlap between them in terms of individual protein changes or enriched biological processes, we next wished to determine whether proteins from the individual datasets may nonetheless converge upon common canonical pathways (i.e., well-characterised metabolic and cell signalling pathways that have been curated using information from published sources). Similar to the cellular and molecular function analysis, canonical pathway analysis using IPA^®^ found far fewer enriched terms associated with the SMA II dataset compared to SMA I and III (Figure 2D), and a three-way comparison in which the enriched canonical pathway annotations across each SMA type were ranked by absolute *p*-value found very little overlap of enrichment per comparison (Figure 2E). Of the enriched canonical pathways identified, none were statistically significant across each of the three SMA datasets (Figure 2D), but several were significantly enriched across two of three datasets, with at least one differentially expressed protein from the third having been associated, including mTOR signalling (SMA I (*p* = 9.01 × 10^−9^) and SMA III (*p* = 4.55 × 10^−3^)), regulation of eIF4 and p70S6K signalling (SMA I (*p* = 3.17 × 10^−6^) and SMA III (*p* = 1.9 × 10^−2^)) and eIF2 signalling SMA I (*p* = 1.64 × 10^−5^) and SMA II (*p* = 9.28 × 10^−3^) (Figure 2E). Protein ubiquitination was also enriched across SMA I (*p* = 4.52 × 10^−4^) and SMA II (*p* = 1.95 × 10^−3^) but just missed the statistical cut-off for significance in SMA III (*p* = 5.62 × 10^−2^) (Figure 2E).

**Figure 3:**
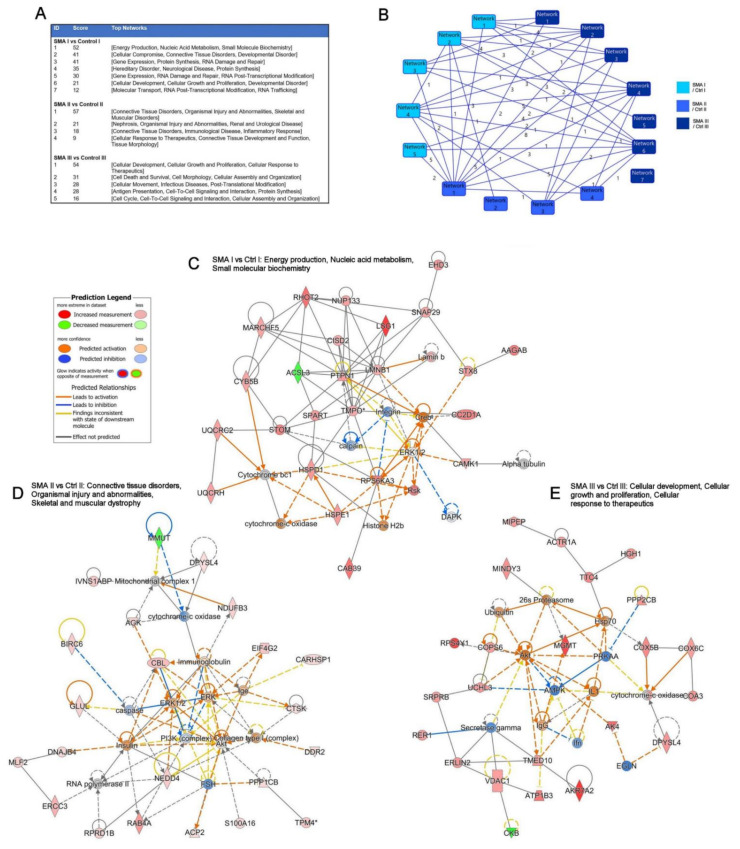
Network analyses of the differentially expressed proteins from each SMA type using IPA^®^. (**A**) Table showing the networks associated with the differentially expressed proteins for each SMA type. The networks are scored based on the number of Network Eligible molecules they contain, with higher scores reflecting a lower probability that the molecules are present by chance. (**B**) Illustration of the interconnectivity of the networks identified for each SMA type, with numbers reflecting the overlap of specific proteins assigned to each network. Schematics of the highest scoring networks associated with the differentially expressed proteins for SMA I (**C**), SMA II (**D**) and SMA III (**E**) using the top 35 molecules for each dataset. Lines represent the biological relationship between two nodes, with direct interactions illustrated as a solid line and indirect interactions as a dotted line.

Subsequent network analysis using IPA^®^ identified several networks with which the differentially expressed proteins from each SMA comparison were associated (Figure 3A), again showing some—but limited—overlap in terms of the specific proteins assigned to each network (Figure 3B). The highest scoring networks for each comparison were associated with energy production, nucleic acid metabolism and small molecular biochemistry biological terms for SMA I (Figure 3C); connective tissue disorders, organismal injury and abnormalities, and skeletal and muscular dystrophy terms for SMA II (Figure 3D); and cellular development, cellular growth and proliferation, and cellular response to therapeutics terms for SMA III (Figure 3E).

### 2.3. Relevance of SMA I-III Fibroblast Proteomic Data to Other Biological Datasets

Using IPA^®^ Analysis Match, we next examined the degree of overlap between the proteomic datasets generated from the SMA Type I, II and III fibroblasts with those from previous SMA studies and studies of neurological conditions. The analysis focused on other datasets with skin or peripheral blood as the tissue of interest since this may reveal insights into the biological relevance of using fibroblasts to study disease mechanisms or to identify disease biomarkers. The term “skin” was used so that studies using fibroblasts would be included. Skin and peripheral blood are tissues that are easier to access and are less invasive for patients than CSF or biopsies from other tissues for the identification and validation of SMA-relevant biomarkers. For this study, outputs relating to Canonical Pathways, Causal Networks, and Diseases and Functions with a minimum positive z-score of 50% were included.

Analysis Match (IPA) applied to the current fibroblast datasets identified a study using SMA Type I fibroblasts [27] that overlapped with a z-score of 60.3 (*p* = 2.00 × 10^−4^) for Canonical Pathways with the current SMA I dataset (Supplementary Materials (Figure S1A)), whilst the current SMA III dataset was found to have a positive overlap (91.29; *p* = 4.86 × 10^−8^) when Canonical Pathways were considered (Supplementary Materials (Figure S1B)) with a study using fibroblasts to investigate neurologic manifestations [28]. No studies within the IPA registry made the 50% z-score cut-off when Causal Networks were considered against either the SMA I or SMA III datasets. In contrast, when the category Diseases and Functions was considered for the SMA I dataset, 11 datasets made the cut-off (z-scores from 52.52 to 66.95; *p* = 6.52 × 10^−12^–8.25 × 10^−27^) with seven datasets utilizing peripheral blood relating to individuals with multiple sclerosis (MS) and four datasets using skin from individuals with amyotrophic lateral sclerosis (ALS; *n* = 1), SMA I (*n* = 1) and neurologic manifestations (*n* = 2) (Supplementary Materials (Figure S1C)). For the SMA III dataset, 26 datasets demonstrated an overlap greater than the 50% z-score cut-off (z-scores ranging from 51.64 to 73.03; *p* = 5.42 × 10^−7^–1.87 × 10^−15^) for the Diseases and Functions outputs, with 20 involving peripheral blood and six using skin (Supplementary Materials (Figure S1D)—top 20 Diseases and Functions). Datasets involving skin investigated familial dysautonomia (*n* = 1), neurologic manifestations (*n* = 2), spinocerebellar ataxia type 6 (*n* = 1) and SMA I (*n* = 2), whilst datasets using peripheral blood studied MS (*n* = 17), Phelan-McDermid syndrome (*n* = 2) and spinocerebellar ataxia type 6 (*n* = 1). No matches with a z-score of 50% or more were made between the curated analyses within IPA and the SMA II fibroblast data for Canonical Pathways, Causal Networks or Diseases and Functions. The only positive overlap was found between four studies in Causal Networks (all z-scores = 20; all *p* = 0.004), with two studies involving skin (one each of SMA I and atypical deletion Williams syndrome) and two studies involving peripheral blood (one each of MS and Friedreich ataxia).

### 2.4. Expression Clustering Analysis Identifies Molecular Profiles That Discriminate and/or Correlate between Different SMA Severities

**Figure 4:**
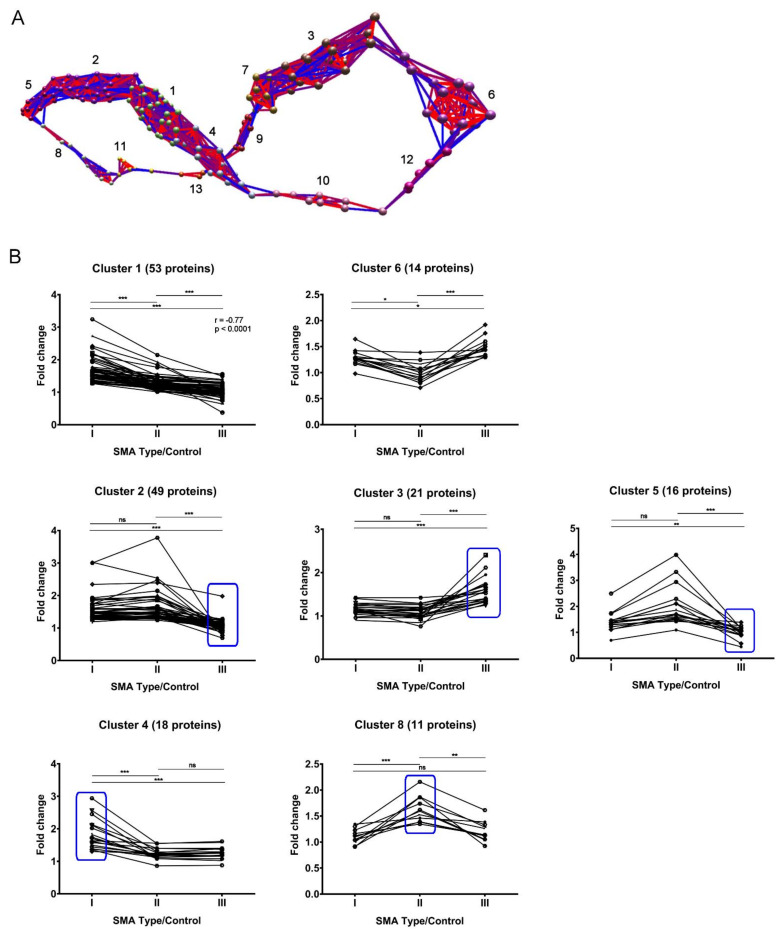
Output following BioLayout Express 3D software analysis of proteins that met the criteria for differential expression in at least one SMA type. (**A**) The complex pattern recognition in BioLayout Express 3D identified 13 different protein expression clusters across the three SMA types. (**B**) Graphs illustrating some of the protein clusters generated in the BioLayout Express 3D analysis. The number of proteins associated with each cluster is given in parentheses. Each point represents a fold change in protein expression relative to age-matched controls, with connecting lines illustrating the trends between SMA I, II and III. Bars indicate significant differences between each SMA type, with * *p* < 0.05; ** *p* < 0.01 and *** *p* < 0.0001 (ns—not statistically significant). In cluster 1, the Spearman rank (r) and associated *p*-value are given for the linear trend in protein fold change between the SMA types. Clusters in which the protein fold changes in one SMA type are significantly different to the other two are highlighted by a blue box.

Having established that there may be potential to identify disease biomarkers from fibroblast samples, we next wished to determine whether protein expression trends are identifiable across the SMA I-III proteomic datasets that discriminate and/or correlate between the different severities. Though it would not be appropriate to quantitatively compare the degree of pathway enrichment across the three SMA fibroblasts datasets in instances where convergence upon common pathways was found, the apparent gradient of enrichment for some pathways (e.g., “EIF2 signaling”; Figure 2E) raised the possibility that protein expression trends across the different types of SMA may have been lost due to the strict filtering criteria applied to the proteomic data. Thus, to identify and visualise protein expression trends that may be present across the three datasets, the individual proteins that met the criteria for differential expression in at least one SMA type were tracked along with the levels found in the remaining SMA types using BioLayout Express 3D software, which enables complex pattern recognition and the subsequent generation of a visual representation of the data based on relative protein abundance [29]. Thirteen clusters were identified according to similarities in their relative expression across the SMA I, II and III datasets (Figure 4A, Supplementary Materials (Figure S2) and Supplementary Table S5). One of these clusters, cluster 1, grouped 53 proteins with expression trends that correlate with SMA severity, having demonstrated both a strong correlation (r = −0.7708, *p* < 0.0001) and statistically significant differences between each SMA type corrected for control levels, i.e., SMA I vs. SMA II and SMA I vs. SMA III (both *p* < 0.0001) and SMA II vs. SMA III (*p* < 0.001) (Figure 4B and Supplementary Table S6). In cluster 6, 14 proteins had expression fold changes that were significantly different between each SMA severity group adjusted for age-matched control levels, with a general trend towards reduced protein expression in SMA II compared to SMA I and SMA III (Figure 4B and Supplementary Table S6), i.e., SMA I vs. SMA II (*p* = 0.023), SMA I vs. SMA III (*p* = 0.03) and SMA II vs. SMA III (*p* < 0.0001). Three separate profiles were identified from the remaining clusters based on statistically significant differences between comparisons, allowing for the identification of protein candidates that discriminate one SMA severity type from the other two types (Figure 4B and Supplementary Table S6). Cluster 2, cluster 3 and cluster 5 incorporated 49, 21 and 16 proteins, respectively, with expression changes that were significantly different in SMA III compared to SMA I and II (again adjusted for control levels), i.e., clusters 2 and 3: SMA I vs. SMA III and SMA II vs. SMA III (both *p* < 0.0001); cluster 5: SMA I vs. SMA III (*p* = 0.0098) and SMA II vs. SMA III (*p* < 0.001). Cluster 4 grouped 18 proteins with expression changes that were significantly different when adjusted for controls in SMA I compared to SMA II and III, i.e., SMA I vs. SMA II and SMA I vs. SMA III (both *p* < 0.0001), and cluster 8 grouped 11 proteins with expression changes compared to controls that were significantly different in SMA II compared to SMA I and III, i.e., SMA I vs. SMA II (*p* < 0.0001) and SMA II vs. SMA III (*p* < 0.0042). Four of the remaining five clusters grouped proteins that showed significantly different expression changes in one SMA type compared to just one other (Supplementary Materials (Figure S2) and Supplementary Table S6).

### 2.5. Quantitative Western Blotting Verifies Potential Utility of Fibroblast Biomarkers for Distinguishing SMA Severities

**Figure 5:**
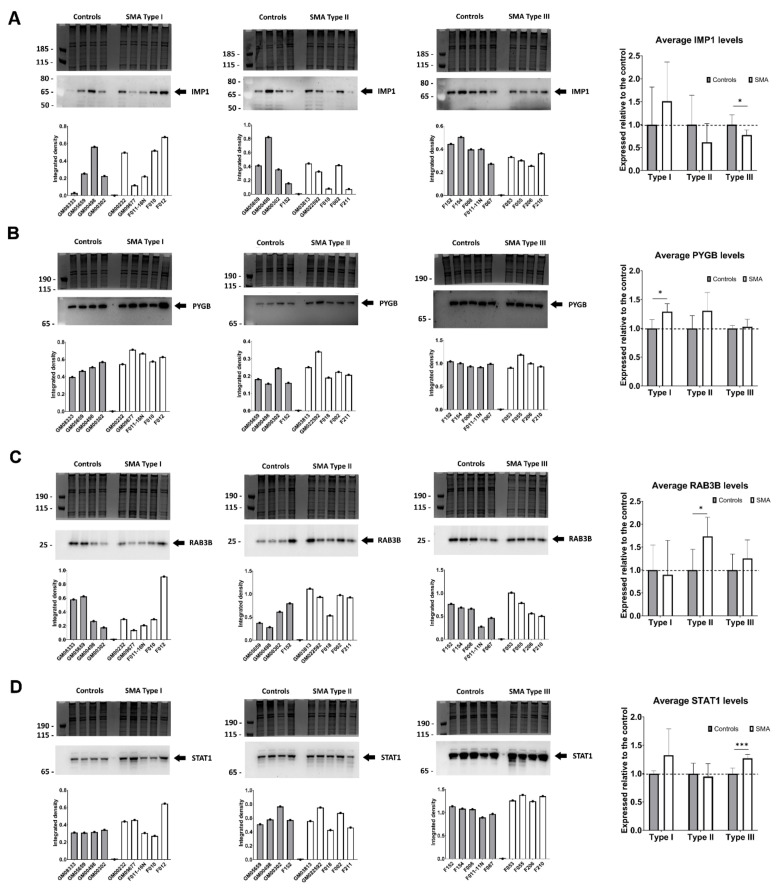
Verification of differential expression of proteins selected from the BioLayout clusters as potential markers for SMA type. Representative Western blots showing levels of (**A**) IMP1, (**B**) PYGB, (**C**) RAB3B and (**D**) STAT1 in each SMA type. Graphs below each Western blot represent the integrated density of antibody-reactive bands/densitometry measurements of a Coomassie-stained gel (i.e., total protein loading control) for individual samples. The summary graphs give the average values for each of the proteins (IMP1, PYGB, RAB3B and STAT1) in each SMA type relative to their respective controls, with a dashed line indicating normalised SMN levels across all control fibroblasts. Error bars show the standard deviation from the mean. *** *p* < 0.001; * *p* < 0.05.

Having identified fibroblast molecular profiles that correlate with SMA severity and those which appear to discriminate SMA I, SMA II and III from each other, four proteins were selected from clusters as potential biomarker candidates for further study. From the SWATH-MS analysis, insulin-like growth factor 2 mRNA-binding protein 1 (IMP1) (cluster 1, Figure 4B) was found to be significantly decreased in SMA III (0.37-fold, *p* = 0.031) whilst significantly increased in SMA I fibroblasts (2.37-fold, *p* = 0.038) compared to their respective controls (Supplementary Table S4). IMP1 expression levels in SMA II compared to controls, meanwhile, did not meet the criteria for differential expression (1.52-fold, *p* = 0.221). Subsequent quantitative Western blot analysis of replicate fibroblast extracts confirmed the significant decrease in IMP1 levels in SMA III compared to age-matched controls (0.77-fold, *p* = 0.042; Figure 5A). As is often the case when analysing human samples; however, consensus could not be reached on IMP1 differential expression in SMA I and II due to variability between individuals (1.51-fold, *p* = 0.196 and 0.61-fold, *p* = 0.172, respectively).

Following SWATH-MS analysis, glycogen phosphorylase, brain form (PYGB) (cluster 2, Figure 4B), was found to be significantly increased in SMA I (1.36-fold, *p* = 0.033) but not significantly changed in either SMA II or III when compared to controls (1.32-fold, *p* = 0.364; 1.14-fold, *p* = 0.361 respectively) (Supplementary Table S4). This finding was corroborated by quantitative Western blot analysis, in which a significant 1.29-fold increase was observed when SMA I fibroblasts were compared with age-matched controls (*p* = 0.012) (Figure 5B). PYGB levels in SMA II and III fibroblasts in comparison to their respective age-matched controls were not statistically different in Western blots (*p* = 0.068 and 0.348, respectively) but were comparable to those found in the SWATH-MS analyses (1.30-fold and 1.03-fold, respectively) (Figure 5B).

From cluster 8, ras-related protein Rab-3B (RAB3B) (Figure 4B) was identified from the SWATH-MS analysis as being significantly increased in SMA II fibroblasts compared to controls (1.62-fold, *p* = 0.032), with no significant changes being detected in SMA I or III (0.92-fold, *p* = 0.755; 1.05, *p* = 0.494 respectively) (Supplementary Table S4). Quantitative Western blot analysis verified these findings, with RAB3B levels showing a significant increase in SMA II compared to age-matched controls (1.73-fold, *p* = 0.022) (Figure 5C). Expression levels of RAB3B in SMA I and III fibroblasts were not significantly different to their relevant controls following Western blot analysis (*p* = 0.409 and 0.181, respectively), with the corresponding ratios being comparable to those observed in the SWATH-MS analysis (0.90-fold and 1.25-fold, respectively) (Figure 5C).

Signal transducer and activator of transcription 1-alpha/beta (STAT1), a component of cluster 6 (Figure 4B), was identified by SWATH-MS analysis as having significantly increased expression in SMA III compared to age-matched controls (1.92-fold, *p* = 0.030) whilst levels in SMA I fibroblasts did not quite meet the significance criteria (1.64-fold, *p* = 0.061), and no changes in STAT1 levels were observed in SMA II (1.05-fold, *p* = 0.816) (Supplementary Table S4). Quantitative Western blot analysis confirmed the statistically significant increase in STAT1 expression in SMA III fibroblasts compared to their respective controls (1.27-fold, *p* = 0.0008) and found expression levels in SMA I and II fibroblasts to mirror the trends observed by SWATH-MS (1.32-fold, *p* = 0.096; 0.95, *p* = 0.355, respectively) (Figure 5D).

### 2.6. Transient Transfection of SMA Fibroblasts with EGFP-SMN1 Reduces Levels of RAB3B in Type II Fibroblasts

**Figure 6:**
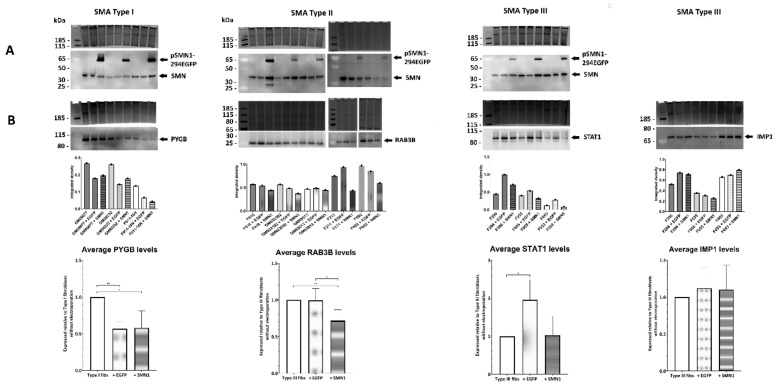
Protein expression changes following transient transfection of SMA Type I, II and III fibroblasts. (**A**) Representative Western blots confirming the presence of plasmids containing full-length human SMN1 (pSMN1-294EGFP) in cells transiently transfected using electroporation. (**B**) Representative Western blots show levels of PYGB, RAB3B, STAT1 and IMP1 following electroporation of the SMA fibroblasts with plasmids containing (+SMN1) or without (+EGFP) SMN. Graphs below each Western blot represent the integrated density of antibody-reactive bands/densitometry measurements of a Coomassie-stained gel (i.e., total protein loading control) for individual samples. The summary graphs give the average values for each of the proteins relative to non-transfected cells. Error bars show the standard deviation from the mean. ** *p* < 0.01; * *p* < 0.05.

To establish whether the expression levels of the potential biomarker candidates identified and verified in Figure 5 could be altered in response to increased SMN levels, fibroblasts from SMA I, II and III were transiently transfected with plasmids containing full-length human SMN1 (pSMN1-294EGFP). Empty plasmids (no SMN) were used as controls for the electroporation process. Overexpression of the full-length SMN protein was seen in all cells transfected with pSMN1-294EGFP at approximately 65 kDa (Figure 6A). Following transfection, levels of PYGB were found to be significantly reduced in SMA I fibroblasts, regardless of whether the cells were electroporated with the empty plasmid (+EGFP) or the plasmid containing SMN1 (Figure 6B), i.e., SMA I vs. SMA I +EGFP (0.57-fold, *p* = 0.007) and SMA I vs. SMA I +SMN1 (0.58-fold, *p* = 0.043), with no significant difference between SMA I +EGFP vs. SMA I +SMN1 (*p* = 0.470). Transient transfection with the plasmid containing SMN1 significantly decreased the levels of RAB3B in the SMA II fibroblasts, whereas transfection with the empty plasmid did not significantly alter RAB3B levels (Figure 6B), i.e., SMA II vs. SMA II +EGFP (no change, *p* = 0.471) and SMA II vs. SMA II +SMN1 (0.72-fold, *p* = 0.007), with significant differences also observed between SMA II +EGFP vs. SMA II +SMN1 (*p* = 0.027). Transfection of SMA III fibroblasts with the empty plasmid elicited an increase in STAT1 levels, whilst transfection with the SMN1-containing plasmid reduced STAT1 levels to those found in non-electroporated SMA III fibroblasts (Figure 6B), i.e., SMA III vs. SMA III +EGFP (1.96-fold, *p* = 0.044) and SMA III vs. SMA III +SMN1 (1.03-fold, *p* = 0.937), with no significant difference between SMA III +EGFP vs. SMA III +SMN1 (*p* = 0.057). In the transiently transfected Type III fibroblasts, no change in IMP1 levels was observed irrespective of whether the cells were electroporated with the empty plasmid or the plasmid containing SMN1 (Figure 6B), i.e., SMA III vs. SMA III +EGFP (1.12-fold, *p* = 0.273), SMA III vs. SMA III +SMN1 (1.10-fold, *p* = 0.327) and no significant difference between SMA III +EGFP vs. SMA III +SMN1 (*p* = 0.431).

## 3. Discussion

The focus of this study was to determine the molecular consequences of reduced SMN levels in fibroblasts from patients with different severities of SMA. Using a quantitative proteomic approach, dysregulated proteins were identified, and their likely impacts on molecular pathways were determined. Interestingly, there was very little overlap between the proteomic profiles of the different SMA types, with not one single protein being significantly altered across all types, resulting in a lack of conserved molecular responses to reduced SMN levels with SMA severity. Of note was the significantly greater variability in the proteomic profile of the SMA II dataset compared to the SMA I and III datasets, even though these fibroblasts were from patients with the same *SMN2* copy number. Previously, differences in the age of fibroblast donors have been shown to impact protein expressed in vitro [30], and other studies have shown age to impact the proteomic profile of plasma samples [31,32]. Whilst all of the SMA I fibroblasts were from infants and all SMA III fibroblasts were from adults (minimum age 17 y), the SMA II fibroblasts were from both infant and adult patients. Although we were careful to include a similar range of age-matched controls, it is possible that this age range, encompassing different stages of life, was a contributing factor to the variance seen between SMA II samples. We cannot rule out the possibility of proteomic changes having occurred from adaptation to culture conditions, but the finding of consistent differences between cell lines of the same types compared to their respective controls suggests that these differences are reflective of SMA severity. In addition, we cannot exclude the possibility of bias in the results due to an unequal number of age-matched controls being used (i.e., four for SMA I and II and five for SMA III). The variance in the SMA II samples could also be explained by the definition of the clinical classification in the three different types, as SMA II may include patients with a broader spectrum of symptoms. The clinical phenotype remains the most discerning factor for SMA type classification, but for future studies, sub-groups of similarly aged individuals within each SMA type may need to be established. Typically, genetic modifiers of SMN expression [33] and modifiers of SMA severity, such as overexpression of plastin 3 (PLS3) and reduced levels of neurocalcin delta (NCALD), result in the absence of any SMA phenotype despite individuals lacking *SMN1* and carrying only 3–4 *SMN2* copies [14,34,35]. A recent study has shown that variants of PLS3 may be present in some SMA patients without influencing the PLS3 expression level and causing a detectable phenotypic change [36]. Indeed, the mechanism for PLS3 overexpression or NCALD downregulation is still unknown, and future work will be useful to determine the reason on the genomic level, as this may have implications for therapy development and biomarker studies.

SMN levels, although reduced in each SMA type compared with age-matched controls, did not correlate with SMA severity. A small, earlier study [37] showed lower SMN protein levels in fibroblasts and lymphoblasts from one SMA I patient in comparison to those from SMA II and III patients (*n* = 1 and 4, respectively), but our findings concur with a larger study of SMN protein expression in patient fibroblasts (SMA I, *n* = 5; SMA II, *n* = 19; and SMA III, *n* = 16) and blood (SMA I, *n* = 18; SMA II, *n* = 60; and SMA III, *n* = 52) [38], plus a biomarker study in which SMN levels in whole blood samples (SMA I, *n* = 17; SMA II, *n* = 49; and SMA III, *n* = 42) were determined [26]. Both studies found SMN levels to be decreased in SMA patients but were unable to stratify patients according to clinical SMA type based on these SMN levels.

Surprisingly, not a single protein was found to be significantly altered across all SMA types, and only a small proportion (14 out of 232) were significantly changed in two SMA types. Few studies have investigated the proteomic profiles of cells from patients with differing SMA severity, but a previous transcriptional study did find 33 genes in muscles from SMA I patients (Type I; *n* = 4;) and 10 genes in muscles from patients with SMA III (*n* = 5) to be significantly altered when compared to control samples [39]. Changes in gene expression do not always relate to altered protein expression [40], but one gene (S100A6) in the transcriptomic study, found to be upregulated in SMA III muscle [39], showed upregulation here in the SMA III fibroblast dataset (1.40-fold increase compared with control cells) with a *p*-value of 0.077. Other differentially expressed genes (NNMT, CYC1, DES, CHCHD3 and ARL6IP5) in the transcriptomic study were also detected in the fibroblast dataset, but none of the changes were statistically significant.

Enriched molecular and cellular processes with which the dysregulated proteins from each SMA type were associated also showed very little overlap, except for necrosis and autophagy for all three types and the metabolism of protein for SMA I and III. Necrosis has been noted as a process that occurs in displaced motor neurons in SMA I and II patients in the later disease stages [41], whilst tissue necrosis was detected in a cohort of SMA patients [42] and in the *Smn^2B^*^/−^ mouse model of SMA [43]. SMN is known to have a key role in protein homeostasis, and its role in autophagy has been scrutinised by several studies (reviewed in [44]), but the interaction between SMN and autophagy remains unresolved, with current findings suggesting that autophagy can be increased or decreased in SMA. The biological processes of prominence in the SMA I fibroblasts are typical of those already associated with SMA, perhaps reflecting the focus of previous research on severe forms of SMA. Energy production is related to mitochondrial dysfunction, which has been demonstrated in studies involving induced pluripotent stem cells (iPSCs) differentiated from the fibroblasts of SMA I patients into spinal motor neurons [45], in primary murine motor neurons [46], in SMA mouse models [47] and in muscle biopsies from SMA I, II and III patients [48], with impaired mitochondrial biogenesis being more prominent in SMA I and least important in SMA III muscles. Ubiquitin-dependent pathways have been shown to be impaired in several models of SMA [49], and although the mechanisms triggered by ubiquitination in RNA damage and repair are not yet fully understood, it is becoming clear that ubiquitination contributes to the degradation of aberrant mRNA and nascent peptides and to ribosome rescue (reviewed by [50]). Evidence for altered gene expression in SMA, which impacts protein synthesis, is substantial (reviewed in [44]). A study using shRNA against *Smn* in rat primary neuron cultures, neurons from a severe mouse model of SMA (*Smn*^−/−^; *SMN2^tg^*^/*0*^) and fibroblast cell lines derived from SMA I patients also provides evidence for impaired de novo protein synthesis efficiency related to reduced mTOR activity in SMA [51]. Evidence is also building for the role of SMN in small nucleolar ribonucleoprotein (snoRNP) assembly and metabolism [44], and as snoRNPs are associated with RNA post-transcriptional modification, this may explain the presence of this biological process in the SMA I fibroblast dataset.

Biological processes impacted in the SMA II fibroblast dataset included carbohydrate metabolism, cellular assembly and organisation, cell morphology, small molecule biochemistry and nucleic acid metabolism. Carbohydrate metabolism has been highlighted in two recent reviews [52,53], with the former highlighting that impairment of glucose metabolism was initially realised in SMA patients with mild to intermediate SMA. Both cellular assembly and organisation and cell morphology have been shown to be impacted in SMA, with evidence of defects in neurite extension, growth cone formation and microtubule formation (reviewed in [44]). Nucleic acid metabolism and small molecule biochemistry share several functions, such as the synthesis of AMP, ADP-D-ribose and purine, and reflect the impact of SMN on metabolic processes [53] and spliceosome function [54].

From the SMA III dataset, cellular movement, development and function, and maintenance included some functions that were relatable to SMA, such as depolarisation of mitochondria, cellular homeostasis and autophagy (reviewed in [44]), but these functions had a tendency to be upregulated. In contrast, post-translational modification included functions not typically associated with SMA, such as phosphorylation of L-tyrosine and both the cleavage and activation of glycoprotein. Cell death and survival included functions such as necrosis and apoptosis, but also an overall positive z-score for cell viability.

The set of networks identified for each SMA type are reflective of the functions and pathways already highlighted above for the different SMA severities. Networks associated with the SMA I fibroblasts show the greatest similarity to findings from other SMA studies and include the network with the terms hereditary disorder and neurological disease. Networks associated with SMA II fibroblasts have a greater emphasis on connective tissue disorders and include nephrosis, which has previously been described in SMA I patients [10]. The networks for the SMA III fibroblasts have a greater emphasis on cellular functions. Although few molecular and cellular pathways and processes are common to all three SMA types, some distinct pathways and processes were identified, especially in the SMA III fibroblasts, that may prove suitable for further investigation to enhance treatment outcomes for these patients. Even though there was greater variation in the data from SMA II fibroblasts, there was still some overlap with the SMA I dataset, indicating that the biological processes in the two more severe forms of SMA may be more closely linked.

None of the enriched canonical pathways identified were statistically significant across all of the three SMA datasets, but several were significantly enriched across two of three datasets, with at least one differentially expressed protein from the third having been associated, including mTOR signalling, regulation of eIF4 and p70S6K signalling, eIF2 signalling and protein ubiquitination. The association of these pathways with SMA is supported by findings in previous studies, where low levels of SMN have been implicated in mTOR signalling [51,55,56], regulation of eIF2 [57] and eIF4 signalling [39], protein ubiquitination [49], mitochondrial dysfunction [45,46,47,48], signalling by Rho family GTPases [58,59] and semaphorin signalling in neurons via increased cleavage of PlexinD1 in SMA [60]. Future targeted experiments will be required, however, to verify and understand the involvement of specific proteins and pathways in less severe forms of SMA. In addition, knowledge of which pathways are implicated throughout stages of disease development in different SMA severities will be important for the development of non-SMN-focused therapies. The lack of molecular and pathway overlap identified across SMA I, II and III in this study further highlights the importance of these considerations, which may lead towards the requirement for a tailored approach to therapy design.

To further investigate the relevance of the current datasets and to determine the possible utility of the proteomic changes as prognostic or treatment efficacy biomarkers, the datasets were compared to other published studies of SMA or similar neurological conditions using Analysis Match within IPA. Only studies of skin (fibroblasts) or peripheral blood were included, as obtaining these tissues is less invasive than taking CSF samples or biopsies of internal organs for patients. The finding of a substantial overlap in canonical pathways between the current SMA I fibroblast dataset and that of a previous study of SMA Type I fibroblasts [27] and a further 11 datasets (for Diseases and Functions) also meeting the filtering criteria provided validation for the SWATH-MS technique used to generate the Type I dataset. Analysis Match was unable to find any similar studies to the SMA II dataset that met the required criteria. This is probably a result of the greater heterogeneity within this dataset. In contrast, Analysis Match of the current SMA III dataset identified a study with a positive overlap [28] in which fibroblasts were used to investigate neurologic manifestations and a further 26 datasets that made the cut-off when Disease and Functions were considered, of which two involved fibroblasts from SMA patients. These findings further support the use of SMA fibroblasts as a biologically relevant source of material to aid in the identification of disease biomarkers, since their proteomic profiles overlap with profiles detected in studies of peripherally accessible tissue and biofluids from SMA and other neurological conditions.

From the BioLayout Express3D analysis, IMP1 was chosen as a candidate for discriminating both Types I and III SMA fibroblasts, as the SWATH-MS data showed an increase in Type I fibroblasts and a decrease in Type III, with Western blots confirming the latter. IMP1 is involved in various biological processes, including nervous system development, neuronal stem cell maintenance and mRNA stabilisation and transport. It is actively transported in motor neuron axons, where it associates with SMN in individual granules [61]. When SMN levels are low, IMP1 is also reduced, and it is suggested that, over time, deficient levels of SMN may result in IMP1 instability and degradation [61]. PYGB was significantly increased in just the Type I SMA fibroblasts and is a cytoplasmic enzyme involved in glycogen mobilisation and metabolism. RAB3B is another cytoplasmic enzyme involved in neurotransmitter regulation, exocytosis and protein transport [62] and was found to be significantly increased in just the Type II fibroblasts. The final candidate biomarker was STAT1, a latent transcription factor that translocates to the nucleus when activated following phosphorylation [63], which is involved in processes such as apoptosis, proliferation, differentiation, cell death and growth. STAT1 was found to be significantly increased in Type III fibroblasts and, along with IMP1 and PYGB, is found within axons. Of the four proteins, however, only RAB3B showed a response to increased SMN levels, suggesting that RAB3B expression is SMN-dependent. Although all of the SMA fibroblasts were successfully transfected, the transfection was only carried out at one DNA concentration, and the cells were harvested at one timepoint. It is therefore possible that alternate transfection conditions may produce different outcomes for the proteins under investigation. It is also quite plausible that the dysregulated proteins identified here could be pathologically relevant and/or suitable as putative biomarkers even if their expression is not SMN-dependent. Although *SMN2* copy number sometimes correlates with the SMA phenotype, this is not the case for all SMA patients [14,15], as supported by findings here that there were SMA patients in each severity classification (Types I, II and III) with three copies of SMN2, while a Type III SMA patient was identified with only two copies of SMN2. This raises a question of how other molecules, which may be unrelated to SMN levels or activity, might modify SMA severity. Considerable support for this notion comes from knowledge of the well-characterised SMA disease modifier PLS3, as described above.

Overall, this study has highlighted the utility of using fibroblasts to identify the molecular profiles and pathways associated with different SMA severities. With further work to verify findings, some of these proteins and pathways may form the foundation to elicit biomarkers for SMA research and treatment monitoring or alternative pathways to target for severity-specific treatments. Although the main degradative process in SMA is loss of motor neurons and muscle wasting, SMN is ubiquitously expressed in all cells, and in this and other studies, there is a clear decrease in SMN levels in fibroblasts. This study has highlighted proteomic and, consequently, altered biological processes that can be attributed to a specific SMA severity. In addition, using IPA, the findings of the current study were matched to other research data, demonstrating robust findings that were matched not only to studies carried out on other fibroblasts but also to peripheral blood analyses. A study in which SMA fibroblasts were used to generate induced pluripotent stem (iPS) cells that were subsequently used to generate motor neurons enabled specific analysis of motor neurons affected by SMA [64]. The aim of this study was to establish whether differentially expressed proteins are detectable in each SMA type. Future studies are now warranted to verify the potential of the dysregulated proteins to act as biomarkers to monitor treatment efficacy, similarly to a recent study where the proteomic profiles of fibroblasts from human skin were used to study the molecular aetiology of rare neurological diseases [65].

In conclusion, this work demonstrates that there is a limited core molecular response to reduced SMN levels across the different severities of SMA and opens the “field” up for a more targeted approach to SMA treatment with respect to SMA type. While four proteins from the datasets were verified, and the impact of increasing SMN levels on these proteins was determined, there remains considerable potential for the remaining candidates found within this study to be explored as specific SMA type biosignatures and/or as biomarkers of both SMN-dependent and SMN-independent SMA treatment efficacy.

## 4. Materials and Methods

### 4.1. Patient Cell Lines

Fibroblast cell lines with GM prefixes were obtained from the Coriell Cell Repository (Camden, NJ, USA), whilst those with the prefix F were acquired from the Newcastle MRC Centre Biobank for Rare & Neuromuscular Diseases (Supplementary Materials (Table S1)). Available information was limited to age, gender and clinical phenotype of SMA severity.

### 4.2. Cell Culture

Fibroblast cell lines (five SMA Type I, five SMA Type II, four SMA Type III and nine age-matched controls) were expanded in cell culture (all reagents from Gibco). Cells were seeded at 5 × 10^3^ per cm^2^ and grown to 70–80% confluency in standard fibroblast medium (Dulbecco’s modified eagle medium (DMEM; 31966-021) containing 10% foetal bovine serum (FBS;10270-098), 1% minimum essential medium non-essential amino acids (MEM NEAA; 11140-035) and 1% Penicillin–Streptomycin (PEN-STREP; 15140-122)) in a humified incubator at 37 °C and 5% CO_2_. Cells were passaged using trypsin/EDTA solution (25200-056; Gibco) for 5 min at 37 °C. Cell pellets for mass spectrometry, Western blotting or *SMN2* copy number analysis were stored at −80 °C until required.

### 4.3. Transient Transfection of Fibroblasts Cells

Plasmids containing full-length human SMN [66] fused to a pEGFP-N2 vector backbone (pSMN1-294EGFP; Clontech, Palo Alto, Santa Clara, CA, USA, 2002) and empty plasmids (pEGFP-N2) were kindly donated by Professor Peter Claus. The plasmids were grown in *E. coli* NovaBlue cells, and following purification using an EndoFree Plasmid Maxi Kit (QIAGEN), the resulting plasmid preparations were analysed by agarose gel electrophoresis and quantified via UV spectrophotometry [67]. Transfection was carried out using an Ingenio^®^ Ezporator^®^ Electroporation System (MIR 51000; Mirus, WI, Madison, WI, USA) and adhering to the manufacturer’s instructions. In brief, the fibroblasts were harvested (approximately 1 × 10^6^ cells mL^−1^) and mixed with 250 µL Ingenio^®^ Electroporation solution containing 5 µg of plasmid DNA and then placed into a 4mm electroporation cuvette. The fibroblasts and plasmids were electroporated at 220 V LV (capacitance = 1050 µF), with the actual peak voltage ranging from 200–222 V and the time constant for the delivered pulse varying from 28–38 ms. Following transfection, the fibroblasts were returned and maintained in standard fibroblast medium for 24 h prior to protein extraction.

### 4.4. DNA Extraction

Genomic DNA was extracted from thawed fibroblast cell pellets resuspended in phosphate-buffered saline (PBS) using the DNeasy^®^ Tissue Kit (QIAGEN) protocol for cultured animal cells. In brief, cells were lysed with proteinase K (#19131; QIAGEN; >600 mAU/mL) for 10 min at 70 °C. Ethanol (96–100%) was added to each sample, and the mixture was then applied to a DNeasy spin column. Following centrifugation steps of ≥6000× *g* and subsequent buffer washes to remove contaminants from the bound DNA, DNA was eluted in the buffer provided. dsDNA concentration and purity were determined using the LVis Plate on a FLUOstar Omega microplate reader (BMG LABTECH, Ortenberg, Germany).

### 4.5. SMN2 Copy Number Determination Using Droplet Digital™ PCR (ddPCR™)

*SMN2* copy number was determined from gDNA extracted from the SMA fibroblast cells using ddPCR™ technology and the ddPCR *SMN2* Copy Number Kit (#1863503; Bio-Rad, Watford, UK). Briefly, a master ddPCR reaction mix was prepared as per kit instructions with HaeIII restriction enzyme (10,000 U/mL; New England BioLabs (NEB), Ipswich, MA, USA) and nuclease-free water (NEB). Each gDNA sample was diluted to a concentration ranging between 6 and 35 ng/µL, and 4 µL was added to the ddPCR reaction mix (total 22 µL), as were the positive (2-, 3- and 4-copy *SMN2*) and no-template controls (NTC). Reaction mixtures were then loaded into an Automated Droplet Generator (#10043138) to produce up to 20,000 droplets per well, which were dispensed in a fresh 96-well plate and subsequently sealed using foil seals and a PX1 plate sealer (#1814000). PCR was carried out using the following steps: 10 min at 95 °C; 40 cycles of 30 s at 94 °C, then 1 min at 55 °C; 10 min at 98 °C, then a hold at 12 °C. The sealed ddPCR plate was then placed into a QX100 Droplet Reader (Bio-Rad), and using the QuantaSoft™ Software CNV application, *SMN2* copy number was determined using *RPP30* as the reference gene. Thresholds were set manually between positive and negative droplets for each well.

### 4.6. Protein Extraction for Mass Spectrometry and Western Blot Analysis

Following passage, cell pellets were washed with PBS. Cell pellets for mass spectrometry (MS) analysis were lysed in 8 M urea dissolved in 100 mM ammonium bicarbonate in sterile distilled water plus 2% sodium deoxycholate. Cell pellets for Western blot (WB) analyses were lysed on ice for 15–20 min with an equivalent volume of RIPA buffer (1% tergitol-type NP-40 (NP40; 9016-45-9, ICN Biomedicals, Aliso Viejo, CA, USA), 0.25% deoxycholic acid (D6750; Sigma, St. Louis, MO, USA), 1mM ethylenediaminetetraacetic acid (EDTA; #104245S, BDH), 150 mM sodium chloride (NaCl; BP358-212, Thermo Fisher Scientific, Waltham, MA, USA) and 50 mM Tris-HCl b, pH 7.4 (Tris; BP152-1, Thermo Fisher Scientific)). All samples were sonicated for 10 s and then centrifugated at 13,000 RPM (MSE, Heathfield, UK; MSB010.CX2.5 Micro Centaur) for 5 min at 4 °C to pellet any insoluble material, and their protein concentrations were determined via Pierce™ BCA Protein Assay Kit (#23227; Thermo Fisher Scientific). Absorbance was measured at 562 nm with a FLUOstar Omega microplate reader (BMG LABTECH), and a standard curve was generated from a plot of the average blank-corrected absorbance for each standard versus its concentration.

### 4.7. Preparation of Samples for IDA and SWATH Mass Spectrometry

A 30 μg aliquot of each sample in 8 M urea/100 mM Ammonium Bicarbonate/2% sodium deoxycholate was reduced with Tris (2-carboxyethyl) phosphine (5 mM) at 30 °C for 1 h, followed by alkylation with iodoacetamide (10 mM) at room temperature (RT) for 30 min in the dark. The reaction was quenched with 20 mM DTT. Prior to trypsin digestion, each sample was diluted to 1.5 M urea and then digested with trypsin (1:50; protease:protein) overnight. Samples were acidified to 0.05% trifluoroacetic acid (TFA).

Peptides were desalted by reversed-phase C-18 and then dried and resuspended to a concentration of 1 µg/µL in loading buffer (2% acetonitrile and 0.05% TFA). SWATH-MS was performed on individual samples. In addition, a pool of all samples was prepared and subjected to nanoLC MS/MS analysis (information-dependent acquisition (IDA)-LC-MS/MS). The remaining peptides were pooled and fractionated by high-pH reverse-phase fractionation (XBridge C18 5µm 4.6 × 100 mm column, Waters) into 12 fractions, which were analysed individually in IDA mode.

### 4.8. Mass Spectrometry Data Acquisition

For IDA, a combined sample (1 μg) or a high-pH RP fraction was injected onto a reverse-phase trap (Acclaim Pepmap 100 μm × 2 cm, Thermo Fisher Scientific) for pre-concentration and desalted with loading buffer at 5 μL/min for 10 min on a nanoLC-MS system (Eksigent nanoLC AS-2/2Dplus system coupled to a Triple TOF 5600+ mass spectrometer, both Sciex). The peptide trap was then switched into line with the analytical column (Acclaim Pepmap RSLC 75 μm × 15 cm, Thermo Fisher). Peptides were eluted from the column at a flow rate of 300 nL/min using a linear solvent gradient: linear 2–20% of buffer B (mobile phase A: 2% acetonitrile, 98% water and 0.1% formic acid; mobile phase B: 98% acetonitrile and 0.1% formic acid) over 90 min, linear 20–40% of buffer B for 30 min, linear 40–98% of buffer B for 10 min, isocratic 98% of buffer B for 5 min, linear 98–2% of buffer B for 2.5 min and isocratic 2% solvent buffer B for 12.5 min. The mass spectrometer was operated in IDA top 20 positive ion mode, with 250 and 150 ms acquisition time for the MS1 (*m*/*z* 400–1250) and MS2 (*m*/*z* 230–1800) scans, respectively, and 15 s dynamic exclusion. Rolling collision energy with a collision energy spread of 5 eV was used for fragmentation. The data files were searched using Mascot against the Swissprot database (January 2019), restricted to only proteins from humans, with trypsin as the cleavage enzyme and carbamidomethylation as a fixed modification of cysteines. Note that the iRT peptides were added to this database.

For SWATH-MS, each sample (1 μg) was injected onto the same LCMS set up as above with the same gradient, except that data acquisition was performed in SWATH mode. The method uses 100 variable window widths with a 1 Da overlap, as developed and optimised by Sciex on plasma. Each window has a dwell time of 150 ms to cover the mass range of 400–1250 *m*/*z* in TOF-MS mode, and MS/MS data are acquired over a range of 230–1800 *m*/*z* with the high sensitivity setting and a dwell time of 35 ms, resulting in a cycle time of 3.7 s. The collision energy for each window was set using the collision energy of a 2^+^ ion centred in the middle of the window with a spread of 5 eV. All Mascot searches using the IDA data were exported as a .dat file and assembled into a spectral library in Skyline by associating each peptide with its respective protein. After the SWATH spectra were imported, peaks were reintegrated using the mProphet peak scoring model [68]. Sum total area [69] was then used to determine fold differences in the protein expression of proteins identified by ≥2 peptides between each SMA type and their respective age-matched controls.

### 4.9. Bioinformatics Analysis

Only proteins with a fold change ≥1.25 or ≤0.80 and a *p*-value ≤ 0.05 were included in further analyses. Venn diagrams were generated with InteractiVenn [70], and heat maps were constructed with Prism Version 8.4.3. Differentially expressed proteins that met the criteria specified above were analysed using Ingenuity Pathway Analysis (IPA) software (Ingenuity Systems, Silicon Valley, CA, USA [71]) to explore the cellular and molecular pathways that may have been altered because of expression changes in SMA I-III fibroblasts versus their age-matched controls. A right-tailed Fisher’s Exact Test was used to calculate the *p*-value determining the probability that each cellular and molecular function or canonical pathway assigned to that dataset is due to chance alone, and the final lists of functions and pathways were ranked accordingly to the resulting *p*-value.

The same datasets of proteins were used for network generation in IPA. Each identifier was mapped to its corresponding entry in Ingenuity’s Knowledge Base, and these proteins were overlaid onto a global molecular network developed from information contained in the Ingenuity Knowledge Base. Networks were then algorithmically generated based on their connectivity. The Functional Analysis of each network identified the biological functions and/or diseases that were most significant to the proteins in the network. A right-tailed Fisher’s Exact Test was used to calculate the *p*-value representing the probability that the overlaps reported between the input dataset and the base pathway/network (e.g., all of the constituent components of RhoGD signalling) are due to chance alone. The resulting networks are a graphical representation of the molecular relationships between proteins, where proteins are represented as nodes, and the biological relationship between two nodes is represented as an edge (line). All edges are supported by at least one reference from the literature, from a textbook or from canonical information stored in the Ingenuity Knowledge Base, all supporting an in vivo or in vitro observation of a protein–protein or protein–DNA interaction (as opposed to a merely predicted interaction from in silico experimentation).

Analysis Match builds a signature from the highest-confidence predictions for a set of data and compares it with those of other analyses generated from public gene expression datasets within IPA’s compendium [72]. A signature is generated for Canonical Pathways, Upstream Regulators, Causal Networks, and Diseases and Functions. If the activated and inhibited entities (proteins in this study) strongly overlap, the generated z-scores will approach 100, whilst analyses that are strongly dissimilar will approach −100. Each signature is created through the filtering of all predicted entities of a single type (e.g., Canonical Pathways) so that only those with Fisher’s Exact Test *p*-values ≤ 0.05 and absolute z-scores ≥ 2 are included [71], with entities with the largest positive and negative z-scores being combined to form the signature. Each entity is assigned a sign, depending on the match, and a scoring algorithm evaluates how well the analyses match. IPA also tests whether the overlap between any two signatures is statistically significant by calculating a *p*-value with Fisher’s Exact Test, which takes into account the number of overlapping entities, the number of non-overlapping entities in both sets of datasets, and the total number of entities in all signatures across all analyses that are not in the two signatures being assessed.

### 4.10. BioLayout Express3D

Protein levels across all three SMA types in which proteins met the criteria for differential expression (i.e., identified from more than one peptide and with a statistically significant (*p*-value of <0.05) fold change of ≥1.25 or ≤0.80) in one or more of the SMA types compared to respective age-matched control fibroblasts were imported separately into BioLayout Express3D [29] and clustered based on the relative expression profile across SMA I, II and III. Algorithms in BioLayout Express3D generate a visual network by extrapolating individual protein identifications in the form of separate data points through their relative expression status in SMA I, II and III fibroblasts compared to age-matched controls. This network uses spatial proximity to represent the similarity in the expression profiles of individual proteins across disease types, whereby proteins that follow a similar expression profile, such as a steady decrease in expression from SMA I/control to SMA II/control to SMA III/control, will be clustered together. The resultant visual networks were utilised first to distinguish expression clusters that followed either a general upward or downward trend in expression from SMA I to II to III/control (i.e., clusters closely correlated with clinical severity). Secondly, expression clusters that “peaked” or “troughed” in a particular SMA type/control compared to both other types were isolated as SMA type-specific trends.

### 4.11. Western Blot Analysis

SDS-PAGE, Western blot and immunodetection were carried out as previously described [73]. Protein extracts from each fibroblast cell line were diluted with 2× Laemmli sample buffer (4% sodium dodecyl sulphate (SDS; l4509, Sigma), 10% 2-mercaptoethanol (M3148; Sigma), 20% glycerol (G9012, Sigma), 0.125 M Tris-HCl and bromophenol blue) and adjusted in concentration to match the sample with the lowest protein concentration as determined from the BCA protein assay. Samples were heated for 3 min at 95 °C and subjected to SDS-PAGE electrophoresis using 4–12% Bis-Tris gels (Invitrogen). Gels were stained with Coomassie blue, and protein loading was determined via densitometry measurements using Image J software (1.52a) [74]. If necessary, sample concentrations were further adjusted to ensure individual protein extracts contributed equally to the WB analysis. Samples were subject to separation by SDS-PAGE, after which a portion of the gel was excised and stained with Coomassie blue as an internal loading control for each sample or group of combined samples. Proteins in the remaining gel were transferred to nitrocellulose membranes overnight, blocked with 4% semi-skimmed powdered milk in PBS for 1 h, then incubated with primary antibodies (Supplementary Materials (Table S2)) for 2 h, washed and incubated with the appropriate HRP-labelled secondary antibody (rabbit anti-mouse Ig (DAKO; P0260) or goat anti-rabbit Ig (DAKO; P0488) diluted 1:1000 (at 0.25 ng/mL) for 1 h. Antibodies were diluted as described in the Supplementary Materials (Table S2) in dilution buffer (1% foetal bovine serum, 1% horse serum and 0.1% BSA in PBS with 0.05% Triton X-100); all steps were carried out at RT, and PBS was used for washes. Following incubation with either West Pico or Femto (Thermo Fisher Scientific), membranes were visualised using a Gel Image Documentation system (Bio-Rad). Detected bands were analysed using Fiji software (v1.51; Madison, WI, USA) [75] and normalised to the densitometry measurements observed on the Coomassie-stained gel.

### 4.12. Statistical Analysis

All statistical analyses were carried out using GraphPad Prism version 9.0.0 (121) for Windows, GraphPad Software, San Diego, CA, USA, www.graphpad.com (accessed on 13 April 2021). The coefficient of variance (CV) was calculated for each protein identified by 2 peptides or more for each SMA type and the associated control group. The CV data of each group were checked for normality using the Anderson–Darling, D’Agostino–Pearson, Shapiro–Wilk and Kolmogorov–Smirnov tests, and Kruskal-Wallis with Dunn’s multiple comparisons test was used to establish any significant differences between the CVs of the groups. To determine whether changes in protein expression of SMA types were significantly different to age-matched controls following SWATH-MS analysis, multiple unpaired t-tests with Welch correction were carried out assuming individual variance for each group and applying no correction for multiple comparisons. Western blot densitometry measurements were assessed using unpaired, two-tailed or one-tailed t-tests (as appropriate) with Welch’s correction. Equal standard deviations were not assumed, and significant differences were determined between age-matched controls and their corresponding SMA type. For comparison of the Western blots of non-transfected and transiently transfected cells, paired t-tests were used. For clusters generated from the BioLayout analysis, Kruskal–Wallis incorporating multiple comparisons and corrected with Dunn’s was used to determine significant differences between groups, whilst Spearman Rank was used to identify any significant correlations.

## Data Availability

The mass spectrometry proteomic data have been deposited to the ProteomeXchange Consortium via the PRIDE [76] partner repository with the dataset identifier PXD034596.

## Supplementary Materials

The following supporting information can be downloaded at: https://www.mdpi.com/article/10.3390/cells11172624/s1, Supplementary Materials (Word document containing Tables S1 and S2 and Figures S1–S3) and Supplementary Tables (Excel document containing Supplementary Tables S3–S6).

## Abbreviations

eIF2 and eIF4	Eukaryotic initiation factor 2 and 4
IMP1	Insulin-like growth factor 2 mRNA-binding protein 1
IPA	Ingenuity Pathway Analysis
mTOR	Mammalian target of rapamycin
PYGB	Glycogen phosphorylase, brain form
RAB3B	Ras-related protein Rab-3B
RHO	Ras homologous protein family
SMA	Spinal muscular atrophy
SMN	Survival of motor neuron
STAT1	Signal transducer and activator of transcription 1-alpha/beta
SWATH-MS	Sequential Window Acquisition of all Theoretical Mass Spectra
