# Supplementary material for: The Proteome Signatures of Fibroblasts from Patients with Severe, Intermediate and Mild Spinal Muscular Atrophy Show Limited Overlap
Source: Cells. 2022 Aug 23;11(17):2624. doi: 10.3390/cells11172624 (PMC9454632; doi:10.3390/cells11172624)
Supplement: Supplementary file 1 [file cells-11-02624-s001.zip › Supplementary Materials.pdf]

The Proteome Signatures of Fibroblasts from Patients with Severe, Intermediate and Mild Spinal Muscular Atrophy Show Limited Overlap

Supplementary File

Sharon J Brown[1,2], Rachel A Kline[3,4], Silvia A Synowsky[5], Sally L Shirran[5], Ian Holt[2], Kelly A Sillence[6], Peter Claus[7], Brunhilde Wirth[8], Thomas M Wishart[3,4], Heidi R Fuller[1,2]*

[1] School of Pharmacy and Bioengineering (PhaB), Keele University, Keele ST5 5BG, UK
[2] Wolfson Centre for Inherited Neuromuscular Disease, RJAH Orthopaedic Hospital, Oswestry SY10 7AG, UK
[3] The Roslin Institute and Royal (Dick) School of Veterinary Studies, University of Edinburgh, Midlothian EH25 9RG, UK
[4] Euan MacDonald Centre, University of Edinburgh, Edinburgh EH16 4SB, UK
[5] BSRC Mass Spectrometry and Proteomics Facility, University of St Andrews, St Andrews, KY16 9ST, UK
[6] Bio-Rad Laboratories, Watford WD17 1ET, UK
[7] SMATHERIA gGmbH—Non-Profit Biomedical Research Institute, Hannover, Germany
[8] Institute of Human Genetics, University Hospital of Cologne, University of Cologne, 50931 Cologne, Germany
[9] Center for Rare Diseases, University Hospital of Cologne, University of Cologne, 50931 Cologne, Germany
[10] Center for Molecular Medicine Cologne, University of Cologne, 50931 Cologne, Germany
[11] Institute for Genetics, University of Cologne, 50931 Cologne, Germany

* Correspondence: h.r.fuller@keele.ac.uk; Tel.: +44-(0)1-782-734546

Fibroblast cells used in the study:

Cell line identifier	Biopsy source	Gender	Age when biopsy taken	Clinical diagnosis	Supplier
**Cell lines used in the SMA I vs age-matched controls comparison**					
F012-16N-ML	skin of left upper arm, outer side	F	4 months	SMA I	Originally from University of Cologne (ID ML112) Newcastle MRC Centre Biobank for Rare & Neuromuscular Diseases
F011-16N-ML	skin of left upper arm, outer side	M	4 months	SMA I	Originally from University of Cologne (ID ML107) Newcastle MRC Centre Biobank for Rare & Neuromuscular Diseases
F010-16N-ML	skin of left upper arm, outer side	F	6 months	SMA I	Originally from University of Cologne (ID ML111) Newcastle MRC Centre Biobank for Rare & Neuromuscular Diseases
GM00232		M	7 months	SMA I	Coriell Cell Repository
GM09677		M	2 years	SMA I	Coriell Cell Repository
GM08333	foreskin	M	5 months	Control	Coriell Cell Repository
GM00302		M	10 months	Control*	Coriell Cell Repository
GM05659	chest	M	1 year	Control*	Coriell Cell Repository
GM00498		M	3 years	Control*	Coriell Cell Repository
**Cell lines used in the SMA II vs age-matched controls comparison**					
GM022592		M	1 year	SMA II	Coriell Cell Repository
GM03813	arm	M	3 years	SMA II	Coriell Cell Repository
F002-15N		M	3 years	SMA II	Newcastle MRC Centre Biobank for Rare & Neuromuscular Diseases
F211-14N		F	23 years	SMA II	Newcastle MRC Centre Biobank for Rare & Neuromuscular Diseases
F018-15N		M	25 years	SMA II	Newcastle MRC Centre Biobank for Rare & Neuromuscular Diseases
GM00302		M	10 months	Control*	Coriell Cell Repository
GM05659	chest	M	1 year	Control*	Coriell Cell Repository
GM00498		M	3 years	Control*	Coriell Cell Repository
F152-14N		F	27 years	Control**	Newcastle MRC Centre Biobank for Rare & Neuromuscular Diseases
**Cell lines used in the SMA III vs age-matched controls comparison**					
F053-15N		M	17 years	SMA III	Newcastle MRC Centre Biobank for Rare & Neuromuscular Diseases
F210-14N		F	22 years	SMA III	Newcastle MRC Centre Biobank for Rare & Neuromuscular Diseases
F055-15N		M	44 years	SMA III	Newcastle MRC Centre Biobank for Rare & Neuromuscular Diseases
F206-14N		M	66 years	SMA III	Newcastle MRC Centre Biobank for Rare & Neuromuscular Diseases
F152-14N		F	27 years	Control**	Newcastle MRC Centre Biobank for Rare & Neuromuscular Diseases
F154-14N		F	34 years	Control	Newcastle MRC Centre Biobank for Rare & Neuromuscular Diseases
F008-16N		M	39 years	Control	Newcastle MRC Centre Biobank for Rare & Neuromuscular Diseases
F011-11N		F	45 years	Control	Newcastle MRC Centre Biobank for Rare & Neuromuscular Diseases
F067-16N		F	66 years	Control	Newcastle MRC Centre Biobank for Rare & Neuromuscular Diseases

Table S1: Cell identity, source of biopsy, gender, age of patient when sampled, phenotype and source of cells are listed for the fibroblasts used in this study. *These cells lines were used as age-matched controls in the SMA I and SMA II comparisons. **This cell line was used as an age-matched control in both the SMA II and SMA III comparisons.

Summary of antibodies used for western blotting:

Target protein	Antibody	Supplier & Source	Molecular Weight (kDa)	Dilution
Survival motor neuron (SMN)	MANSMA12; Clone 2E6	Wolfson CIND; Mouse monoclonal	38	1:100
Insulin-like growth factor 2 mRNA-binding protein 1	IMP1 (D33A2); #8482	Cell Signaling Technology; Rabbit monoclonal	64	1:500
Glycogen phosphorylase, brain form	PYGB/M (17B6); sc-51923	Santa Cruz BioTechnology, Inc; Mouse monoclonal	97	1:200
Ras-related protein Rab-3B	Rab 3B (48-K2): sc-81911	Santa Cruz BioTechnology, Inc; Mouse monoclonal	25	1:50
Signal transducer and activator of transcription 1-alpha/beta	STAT1; 10144-2-AP	Proteintech; Rabbit polyclonal	84	1:500

Table S2: Details of target protein, antibody name and associated clone, supplier, source, expected molecular weight and dilution of antibodies used in this study.

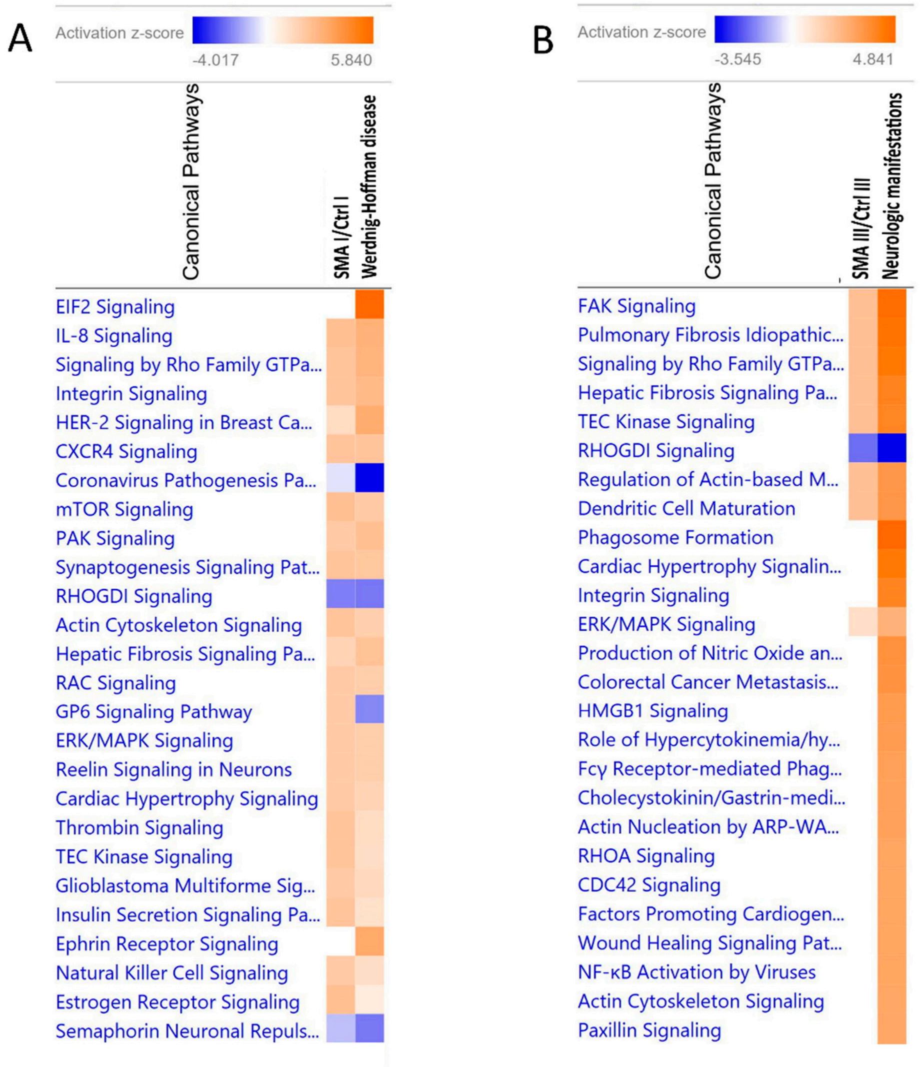
A
Activation z-score
-4.017
5.840
Canonical Pathways
SMA I/Ctrl I
Werdnig-Hoffman disease
EIF2 Signaling
IL-8 Signaling
Signaling by Rho Family GTPa...
Integrin Signaling
HER-2 Signaling in Breast Ca...
CXCR4 Signaling
Coronavirus Pathogenesis Pa...
mTOR Signaling
PAK Signaling
Synaptogenesis Signaling Pat...
RHOGDI Signaling
Actin Cytoskeleton Signaling
Hepatic Fibrosis Signaling Pa...
RAC Signaling
GP6 Signaling Pathway
ERK/MAPK Signaling
Reelin Signaling in Neurons
Cardiac Hypertrophy Signaling
Thrombin Signaling
TEC Kinase Signaling
Glioblastoma Multiforme Sig...
Insulin Secretion Signaling Pa...
Ephrin Receptor Signaling
Natural Killer Cell Signaling
Estrogen Receptor Signaling
Semaphorin Neuronal Repuls...
B
Activation z-score
-3.545
4.841
Canonical Pathways
SMA III/Ctrl III
Neurologic manifestations
FAK Signaling
Pulmonary Fibrosis Idiopathic...
Signaling by Rho Family GTPa...
Hepatic Fibrosis Signaling Pa...
TEC Kinase Signaling
RHOGDI Signaling
Regulation of Actin-based M...
Dendritic Cell Maturation
Phagosome Formation
Cardiac Hypertrophy Signalin...
Integrin Signaling
ERK/MAPK Signaling
Production of Nitric Oxide an...
Colorectal Cancer Metastasis...
HMGB1 Signaling
Role of Hypercytokinemia/hy...
Fcγ Receptor-mediated Phag...
Cholecystokinin/Gastrin-medi...
Actin Nucleation by ARP-WA...
RHOA Signaling
CDC42 Signaling
Factors Promoting Cardiogen...
Wound Healing Signaling Pat...
NF-κB Activation by Viruses
Actin Cytoskeleton Signaling
Paxillin Signaling

C

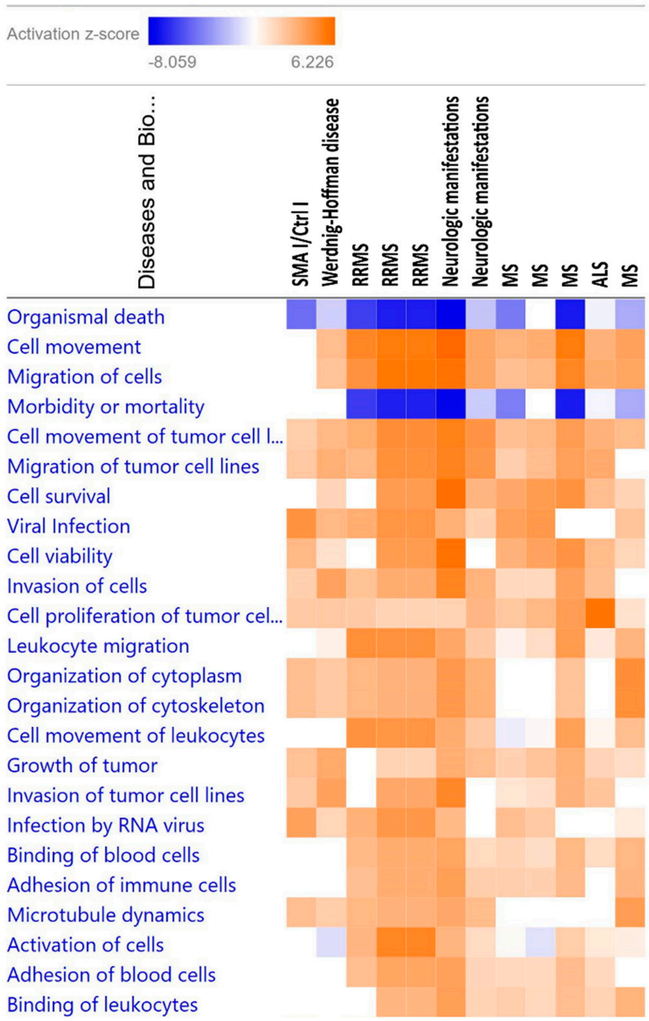

Activation z-score
-8.059
6.226
Diseases and Bio...
SMA I/Ctrl I
Werdnig-Hoffman disease
RRMS
RRMS
RRMS
Neurologic manifestations
Neurologic manifestations
MS
MS
MS
ALS
MS
Organismal death
Cell movement
Migration of cells
Morbidity or mortality
Cell movement of tumor cell l...
Migration of tumor cell lines
Cell survival
Viral Infection
Cell viability
Invasion of cells
Cell proliferation of tumor cel...
Leukocyte migration
Organization of cytoplasm
Organization of cytoskeleton
Cell movement of leukocytes
Growth of tumor
Invasion of tumor cell lines
Infection by RNA virus
Binding of blood cells
Adhesion of immune cells
Microtubule dynamics
Activation of cells
Adhesion of blood cells
Binding of leukocytes

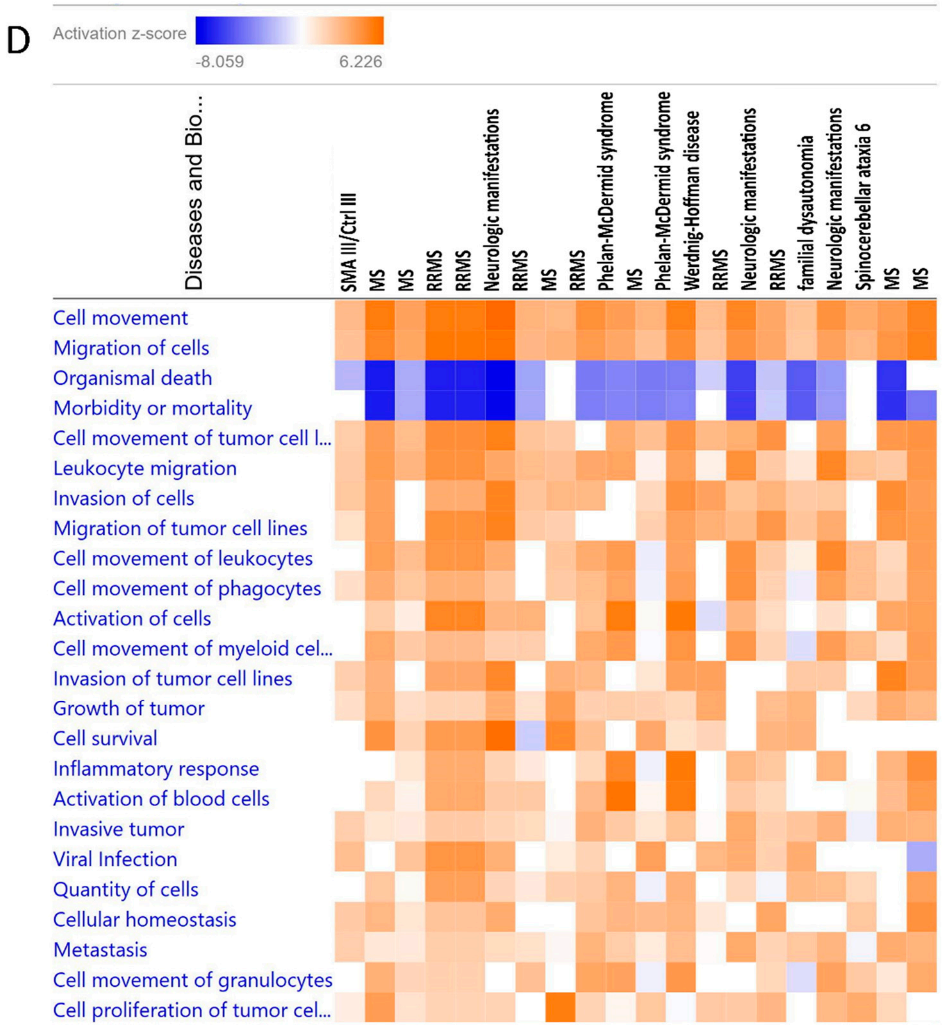

Figure S1: Heat maps using z-scores to illustrate the degree of overlap between proteomic datasets generated in this study with previous SMA studies and studies of neurological conditions with similar outcomes to SMA identified via IPA® Analysis Match. Only signatures with a minimum positive z-score of 50 and using either skin or peripheral blood as the tissues of interest were included. Analysis Match outcomes for canonical pathways identified from (**A**) the SMA I dataset and (**B**) the SMA III dataset and Analysis Match outcomes for diseases & functions identified using (**C**) the SMA I dataset and (**D**) the SMA III dataset. MS = multiple sclerosis; RRMS = relapsing-remitting multiple sclerosis

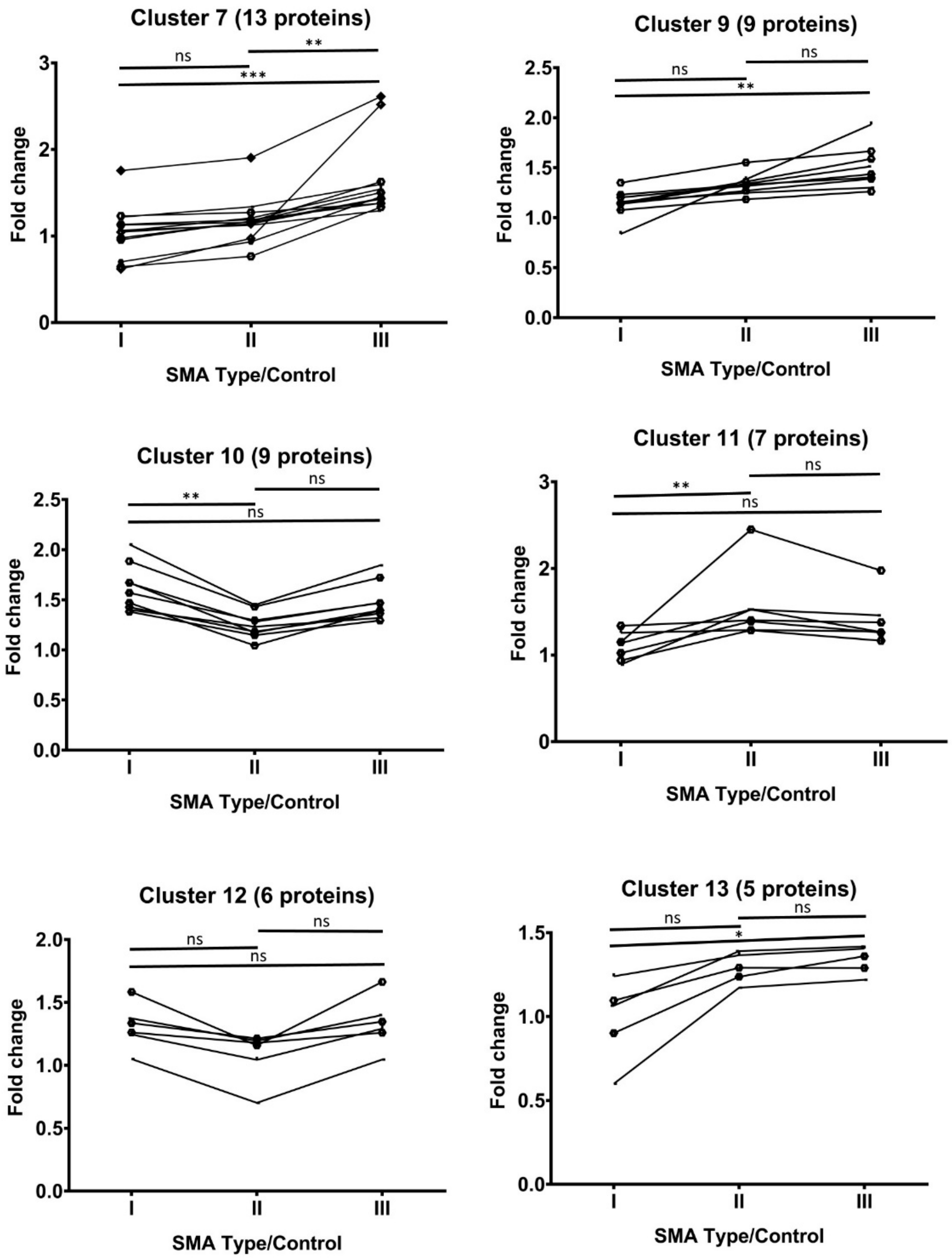

Figure S2: Clusters identified from BioLayout Express 3D analysis of protein expression trends across the three SMA types that met the criteria for differential expression in at least one SMA type (i.e., detected with 2 or more peptides; fold change ≥1.25 or ≤0.80; significant difference of $p \leq 0.05$). Each point represents a fold change in protein expression relative to age-match controls with connecting lines illustrating the trends between SMA I, II and III. Bars indicate level of significance between each SMA type with *$p<0.05$; **$p<0.01$ & ***$p<0.0001$.

	PYBG	STAT1	RAB3B
**Type I SMA**	**Significantly increased**	Potentially increased	Unchanged
**Type II SMA**	Potentially increased	Unchanged	**Significantly increased**
**Type III SMA**	Unchanged	**Significantly increased**	Potentially increased

Figure S3: Table illustrating how the differential expression of PYGB, STAT1 and RAB3B, as determined via SWATH mass spectrometry and confirmed via western blots, relate to SMA severity.
